# Supplementary material for: Overlay databank unlocks data-driven analyses of biomolecules for all
Source: Nat Commun. 2024 Feb 7;15:1136. doi: 10.1038/s41467-024-45189-z (PMC10850068; doi:10.1038/s41467-024-45189-z)
Supplement: Supplementary file 1 — Supplementary information [file 41467_2024_45189_MOESM1_ESM.pdf]

SUPPLEMENTARY INFORMATION: Overlay databank unlocks data-driven analyses of biomolecules for all

Anne M. Kiirikki[1], Hanne S. Antila[2,3], Lara S. Bort[2,4], Pavel Buslaev[5], Fernando Favela-Rosales[6], Tiago Mendes Ferreira[7], Patrick F.J. Fuchs[8,9], Rebeca Garcia-Fandino[10], Ivan Gushchin , Batuhan Kav[11,12], Norbert Kučerka[13], Patrik Kula[14], Milla Kurki[15], Alexander Kuzmin , Anusha Lalitha[16], Fabio Lolicato[17,18], Jesper J. Madsen[19,20], Markus S. Miettinen[2,21,22], Cedric Mingham[23], Luca Monticelli[24,25], Ricky Nencini[1,26], Alexey M. Nesterenko[21,22], Thomas J. Piggot[27], Ángel Piñeiro[28], Nathalie Reuter[21,22], Suman Samantray[12,29], Fabián Suárez-Lestón[10,28,30], Reza Talandashti[21,22], and O. H. Samuli Ollila[1,31,*]

[1]University of Helsinki, Institute of Biotechnology, Helsinki, Finland
[2]Department of Theory and Bio-Systems, Max Planck Institute of Colloids and Interfaces, 14424 Potsdam, Germany
[3]Department of Biomedicine, University of Bergen, 5020 Bergen, Norway
[4]University of Potsdam, Institute of Physics and Astronomy, Potsdam-Golm, 14476, Germany
[5]Nanoscience Center and Department of Chemistry, University of Jyväskylä, Jyväskylä, 40014, Finland
[6]Departamento de Ciencias Básicas, Tecnológico Nacional de México - ITS Zacatecas Occidente, Sombrerete, Zacatecas, 99102, México
[7]NMR group - Institute for Physics, Martin Luther University Halle-Wittenberg, Halle (Saale), 06120, Germany
[8]Sorbonne Université, Ecole Normale Supérieure, PSL University, CNRS, Laboratoire des Biomolécules (LBM), F-75005 Paris, France
[9]Université Paris Cité, UFR Sciences du Vivant, Paris, 75013, France
[10]Center for Research in Biological Chemistry and Molecular Materials (CiQUS), Universidade de Santiago de Compostela, Santiago de Compostela, E-15782, Spain
[11]Institute of Biological Information Processing: Structural Biochemistry (IBI-7), Forschungszentrum Jülich, 52428 Jülich, Germany
[12]ariadne.ai GmbH (Germany), Häusserstraße 3 Heidelberg 69115, Germany
[13]Department of Physical Chemistry of Drugs, Faculty of Pharmacy, Comenius University Bratislava, 832 32 Bratislava, Slovakia
[14]Institute of Organic Chemistry and Biochemistry of the Czech Academy of Sciences, Flemingovo nám. 542/2, Prague, CZ-16610, Czech Republic
[15]School of Pharmacy, University of Eastern Finland, 70211 Kuopio, Finland
[16]Institut Charles Gerhardt Montpellier (UMR CNRS 5253), Université Montpellier, Place Eugène Bataillon, 34095 Montpellier, Cedex 05, France
[17]Heidelberg University Biochemistry Center, 69120 Heidelberg, Germany
[18]Department of Physics, University of Helsinki, FI-00014 Helsinki, Finland
[19]Department of Molecular Medicine, Morsani College of Medicine, University of South Florida, Tampa, Florida 33612, United States of America
[20]Center for Global Health and Infectious Diseases Research, Global and Planetary Health, College of Public Health, University of South Florida, Tampa, Florida 33612, United States of America
[21]Department of Chemistry, University of Bergen, 5007 Bergen, Norway
[22]Computational Biology Unit, Department of Informatics, University of Bergen, 5008 Bergen, Norway
[23]Hochschule Mannheim, University of Applied Sciences, Mannheim, Germany
[24]University of Lyon, CNRS, Molecular Microbiology and Structural Biochemistry (MMSB, UMR 5086), F-69007, Lyon, France
[25]Institut National de la Santé et de la Recherche Médicale (INSERM), Lyon, France
[26]Division of Pharmaceutical Biosciences, Faculty of Pharmacy, University of Helsinki, Helsinki 00014, Finland
[27]Chemistry, University of Southampton, Highfield, Southampton, SO17 1BJ, United Kingdom
[28]Department of Applied Physics, Faculty of Physics, University of Santiago de Compostela, Santiago de Compostela, E-15782, Spain
[29]Institute of Biotechnology, RWTH Aachen University, Worringerweg 3, 52074 Aachen, Germany

[30]MD.USE Innovations S.L., Edificio Emprendia, 15782 Santiago de Compostela, Spain
[31]VTT Technical Research Centre of Finland, Espoo, Finland
[*]samuli.ollila@helsinki.fi

Databank content

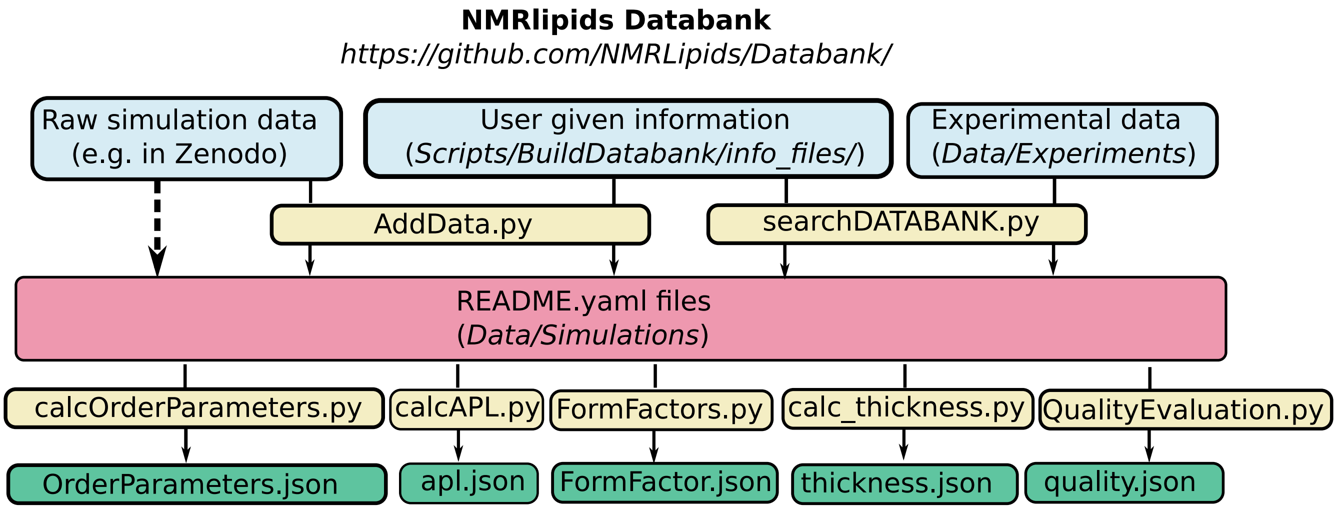

Supplementary Figure 1. Structure of the NMRlipids Databank. Manually added input data (blue boxes) include basic information on the simulation, permanent links to the raw data, and experimental data if available. The databank entries (red box) and analysis results (green boxes), at https://github.com/NMRlipids/Databank/tree/main/Data/Simulations are automatically generated by the computer programs included in the NMRlipids Databank (yellow boxes). Because the raw data are not permanently stored but can be accessed based on the information in the Databank, this connection is marked with a dashed line.

Force field name and references
CHARMM36[1]
Slipids[2–6]
MacRog[7]
Amber Lipid14/17[8,9]
Charmm-Drude[10]
ECClipids[11–13]
GROMOS-CKP[14–16]
Berger[17]
ECC-CHARMM36[18]
Orange[19]
Poger[20]
GROMOS 43A1-S3[21]
GAFFlipid[22]
OPLS3e[23]
Ulmschneider[24]
Chiu Gromos[21]
AMOEBA[25]
OpenFF[26–28]

Supplementary Table 1. List of current force fields used in simulations in the Databank, with references.

Code	Function	Input	Output
**Application layer**			
AreaPerLipidAnd ThicknessCorrelations.ipynb	Analyze correlations between results in the *Databank layer*	Results in the *Databank layer*	Figure 2G in the main text and Supplementary Figure 3
FlipFlop.py	Calculate flip-flop rates of lipids in all simulations	README.yaml	flipflop.dat files at */Data/Flipflops/*
plotFlipFlop.ipynb	Plot how flip-flop rates depend on membrane properties	README.yaml and flipflop.dat files at */Data/Flipflops/*	Figure 4 in the main text
calcMD-PERMEATION.py	Calculate permeation rate of water molecules through bilayers from all simulations	README.yaml	Counting_events.txt files at */Data/MD-PERMEATION/*
calcWATERdiffusion.py	Calculate water lateral diffusion along membrane surface	README.yaml	WATERlateralMSD.xvg files at */Data/WATERdiffusion/*
plotWaterPermeation.ipynb	Plot how water permeation and diffusion depend on membrane properties	README.yaml, Counting_events.txt at */Data/MD-PERMEATION/* and WATERlateralMSD.xvg files at */Data/WATERdiffusion/*	Figure 5 in the main text, and Supplementary Figures 8, and 9.
APLpredictor.ipynb	Predict area per lipid of a membrane based in its lipid composition from machine learning models trained using the NMRlipids databank	README.yaml and apl.dat files	Machine learning models at /Data/APLpredictor/

Supplementary Table 2. Examples of codes that analyze membrane properties from the Databank in an *Application layer* available at https://github.com/NMRLipids/DataBankManuscript/.

key	description	type
DOI	DOI from where the raw data is found	user given (compulsory)
SOFTWARE	Software used to run the simulation (Gromacs, Amber, NAMD, etc.)	
TRJ	Name of the trajectory file found from DOI (trr or xtc for Gromacs, dcd for OpenMM)	
TPR (Gromacs)	Name of the tpr topology file found from DOI for Gromacs simulations	
PDB (OpenMM)	Name of the pdb file found from DOI for OpenMM simulations	
PREEQTIME	Pre-equilibrate time simulated before the uploaded trajectory in nanoseconds.	
TIMELEFTOUT	Equilibration period in the uploaded trajectory that should be discarded in analyses.	
COMPOSITION	Dictionary connecting universal molecule and atom names to the ones used in simulation	
DIR_WRK	Temporary working directory in your local computer.	
UNITEDATOM_DICT	Information for constucting hydrogens for united atom simulations using buildH program[29]. Empty for all atom simulations.	
TYPEOFSYSTEM	Lipid bilayer or something else	
PUBLICATION	Give reference to a publication(s) related to the data	user given (optional)
AUTHORS_CONTACT	Name and email of the main author(s) of the data	
SYSTEM	System description on free text format	
SOFTWARE_VERSION	Version of the used software	
FF	Name of the used force field	
FF_SOURCE	Source of the force field parameters, e.g, CHARMM-GUI, webpage, citation to a publication	
FF_DATE	Date when force field parameters were accessed on the given source (day/month/year)	
FFmolename	Molecule specific force field information, e.g., water model with FFSOL and sodium parameters with FFSOD	
CPT	Name of the Gromacs checkpoint file	
LOG	Name of the Gromacs log file	
GRO	Name of the Gromacs gro file	
TOP	Name of top file for Gromacs or psf file for OpenMM	
CRD	Name of crd file for OpenMM	
WARNINGS	Dictionary containing information about unusual features in the trajectory, such as ambiguous atom names, membrane normal not oriented in z-direction, old Gromacs version used	
TRAJECTORY_SIZE	Size of the trajectory file in bytes	automatically extracted data
TRJLENGTH	Lenght of the trajectory (ps)	
TEMPERATURE	Temperature of the simulation	
NUMBER_OF_ATOMS	Number of atoms in the simulation	
DATEOFRUNNIG	Date when added into the Databank	
EXPERIMENT	Potentially connected experimental data	
COMPOSITION	Numbers of lipid molecules in both leaflets and numbers of other molecules are added to the dictionary	
ID	Unique ID number to ease the analyses	

Supplementary Table 3. Keys stored in the README.yaml files of simulations.

key	description
DOI	DOI of the publication related to the experimental data
TEMPERATURE	Temperature of the experiment
MOLAR_FRACTIONS	Dictionary of molar fractions of bilayer components
ION_CONCENTRATIONS	Dictionary of ion concentrations of the system
TOTAL_LIPID_CONCENTRATION	Total concentration of lipid components; if exact concentration is not known, but experiments are performed in excess water, 'full hydration' can be given
COUNTER_IONS	Type of counter ions if present

Supplementary Table 4. Keys stored in the README.yaml files of experiments.

Code	Function	Input	Output
	**Databank layer**		
	**Scripts/BuildDatabank/**		
AddData.py	Create README.yaml from user given information in info.yaml	info.yaml	README.yaml
searchDATABANK.py	Pair simulations with available experimental data	README.yaml files	Updated README.yaml files
QualityEvaluation.py	Quality-evaluate simulations that are paired with experimental data	README.yaml files	[lipid_name]_OrderParameters_quality.json, [lipid_name]_FragmentQuality.json, system_quality.json, FormFactorQuality.json
	**Scripts/AnalyzeDatabank/**		
calcOrderParameters.py	Calculate C-H bond order parameters for all lipids in the simulation	README.yaml files	[lipid_name]_OrderParameters.json
calcAPL.py	Calculate area per lipid as a function of time	README.yaml files	apl.json
calc_FormFactors.py	Calculate x-ray scattering form factors	README.yaml files	FormFactor.json
calc_thickness.py	Calculate membrane thickness	README.yaml files	thickness.json

Supplementary Table 5. List of relevant codes used to build the Databank and perform analyses in the *Databank layer* available at https://github.com/NMRLipids/Databank/.

Processing results in the application layer

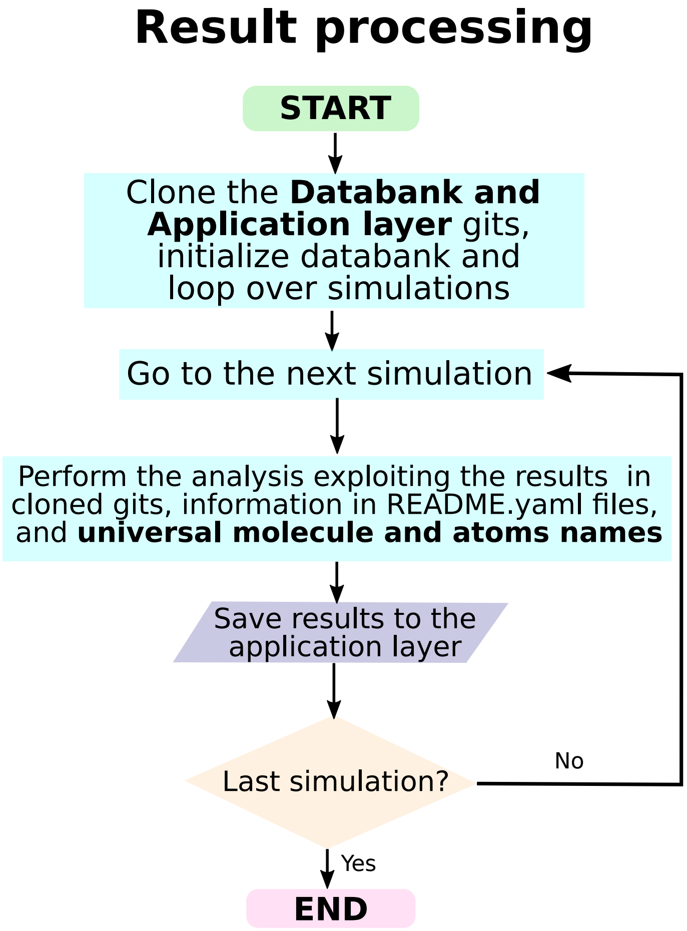

Supplementary Figure 2. Flowchart for accessing results calculated from the NMRlipids Databank and stored to the *Application layer*.

Correlations of area per lipid and thickness with order parameters and form factors

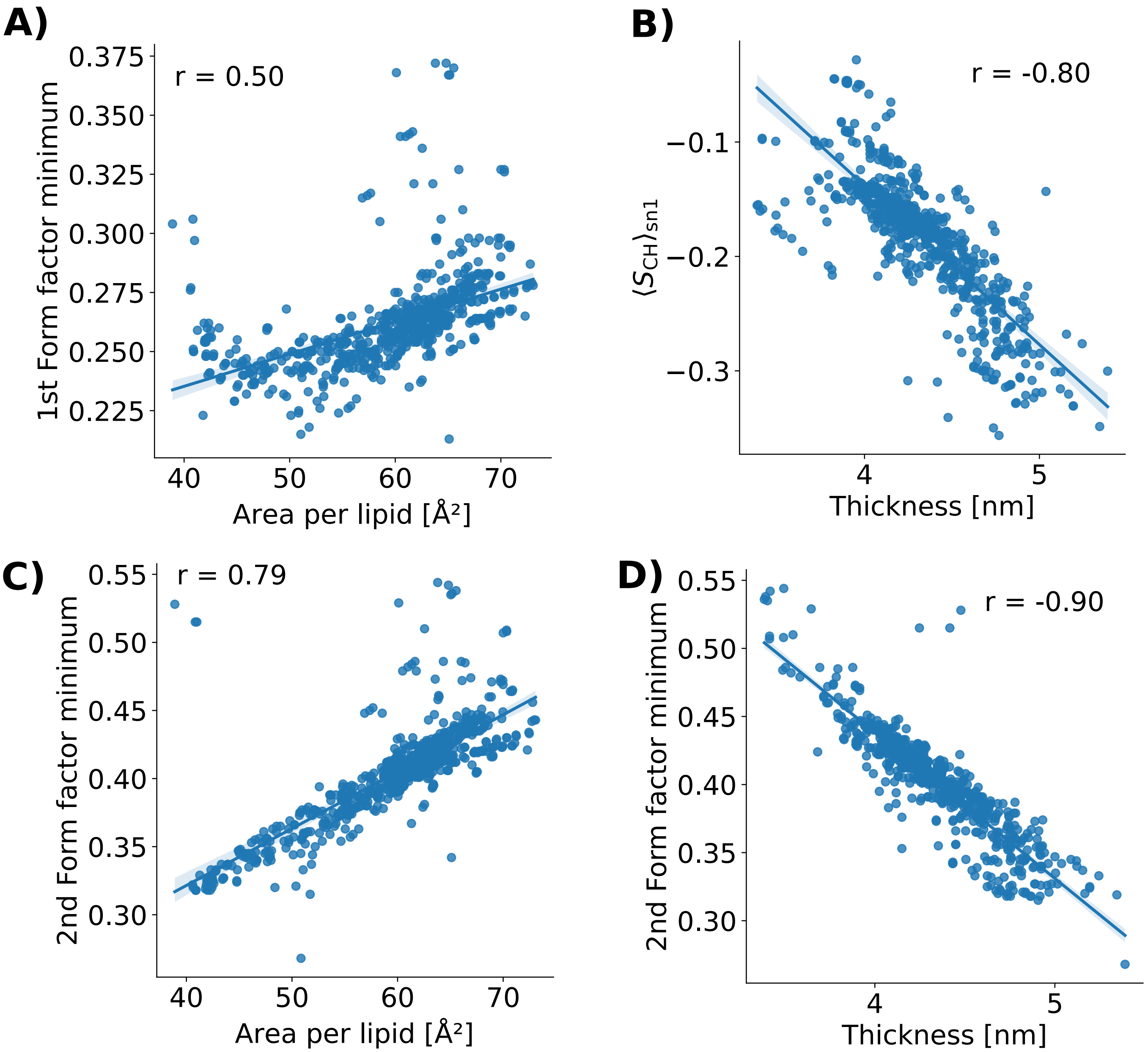

Supplementary Figure 3. Scatter plots and Pearson correlation coefficients, *r*, for the membrane area per lipid with X-ray scattering form factor minima (A and B), and for thickness with the average order parameter of the *sn*-1 acyl chain (B) and with the second minimum from X-ray scattering form factors (D) extracted from the NMRlipids databank. All correlation coefficients have p-value below 0.001.

Dependence of form factor and order parameters on the simulation box size

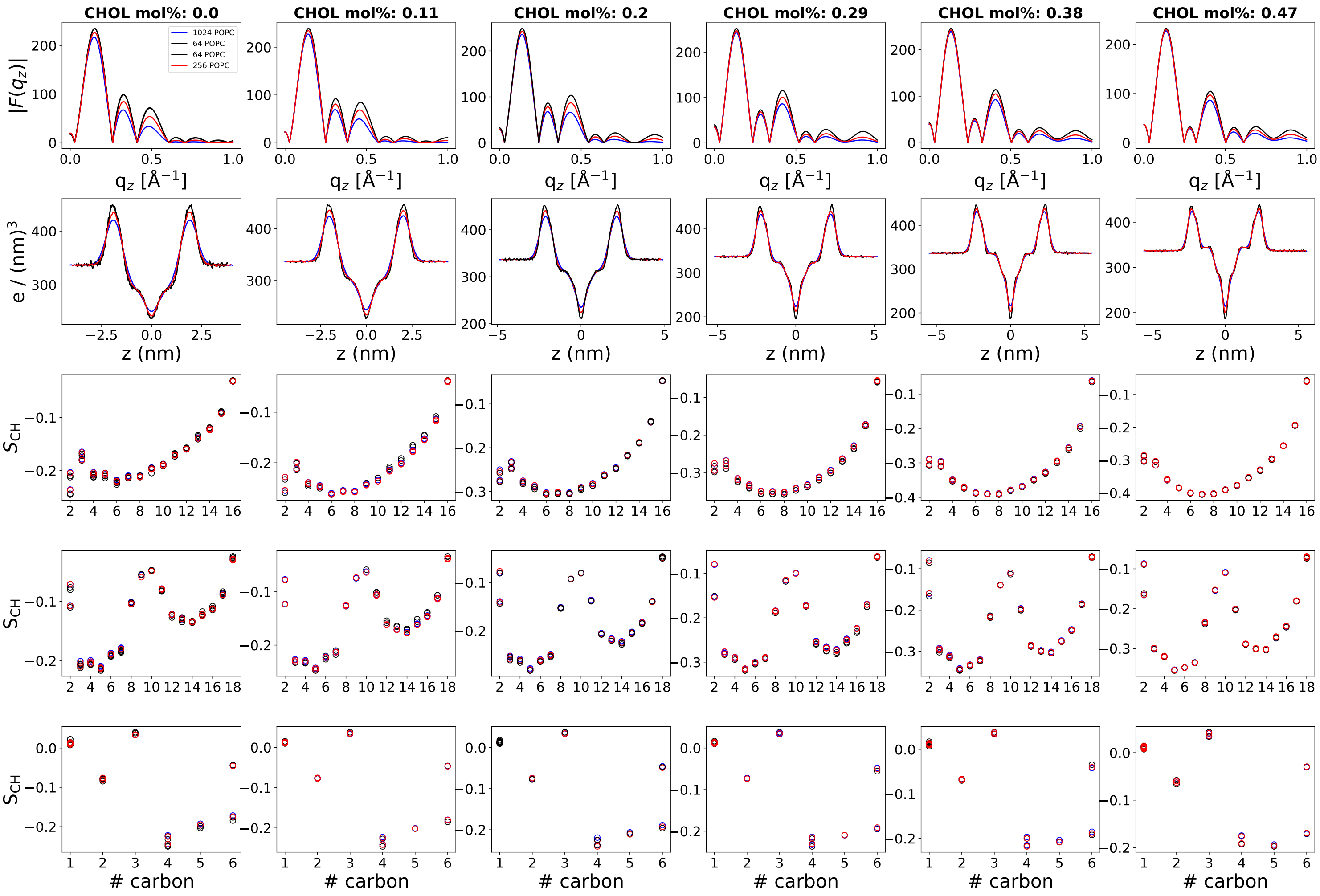

Supplementary Figure 4. Dependence of the form factor $F(q_z)$, the electron density profiles along membrane normal, and the C–H bond order parameters S_{CH} (from top to bottom) on the simulation box size (with different columns showing different cholesterol concentrations). Simulations with 64, 256, and 1024 POPC lipids are from Ref. 30.

Finding the best models for PC and PE mixtures

	P^{tails}	P^{hg}	P^{total}	FF_q	τ_{rel}	Force field	Molecules	Temperature	ID
1	0.86	0.76	0.83	0.15	0.4	OPLS3e	POPC:SOL (200:8859)	300.00	1
2	0.83	0.61	0.76	0.45	7.4	Slipids	POPC:SOL (512:23943)	298.00	617
3	0.81	0.58	0.73	0.45	0.4	Slipids	POPC:SOL (1024:51200)	298.15	696
4	0.73	0.70	0.72	0.65	0.3	MacRog	POPC:SOL (1024:51200)	298.15	658
5	0.73	0.63	0.69	0.55	0.5	MacRog	POPC:SOL (128:5120)	300.00	457
6	0.76	0.54	0.69	0.55	0.4	Slipids	POPC:SOL (256:12800)	298.15	708
7	0.69	0.67	0.68	0.55	0.3	MacRog	POPC:SOL (256:12800)	298.15	675
8	0.71	0.62	0.68	0.55	0.3	MacRog	POPC:SOL (64:3200)	298.15	674
9	0.69	0.54	0.64	0.76	0.4	Slipids	POPC:SOL (64:3200)	298.15	664
10	0.60	0.64	0.61	0.55	7.7	MacRog	POPC:SOL (288:14400)	298.00	63
11	0.58	0.65	0.60	0.55	1.0	ECC-lipids	POPC:SOL (128:6400)	300.00	573
12	0.66	0.47	0.60	0.15	0.2	Lipid17	POPC:SOL (64:3200)	298.15	715
13	0.57	0.60	0.58		0.6	AMOEBA	DOPC:SOL (72:2880)	303.00	742
14	0.60	0.53	0.58	0.25	0.2	Lipid17	POPC:SOL (256:12800)	298.15	657
15	0.86	0.01	0.58	0.95	0.4	Berger	POPC:SOL (256:10342)	300.00	115
16	0.58	0.54	0.57	0.25	0.2	Lipid17	POPC:SOL (1024:51200)	298.15	684
17	0.81	0.02	0.55	1.05	1.7	Berger	POPC:SOL (128:7290)	298.00	497
18	0.49	0.65	0.54	0.76	1.1	ECC-lipids	POPC:SOL (128:6400)	300.00	43
19	0.65	0.29	0.53	0.4	1.3	GROMOS-CKP	POPE:SOL (500:25000)	310.00	400
20	0.78	0.01	0.52	0.1	1.3	Slipids	POPE:SOL (500:25000)	310.00	414
21	0.72	0.10	0.51		0.7	Slipids	POPE:SOL (336:13460)	310.00	29
22	0.71	0.10	0.51		0.8	Slipids	POPE:SOL (336:13460)	310.00	74
23	0.66	0.16	0.50	0.4	0.2	ECClipids	POPS:SOL:SOD (72:3600:72)	298.00	443
24	0.38	0.67	0.48	1.26	0.7	CHARMM36	POPC:SOL (256:9767)	300.00	558
25	0.38	0.67	0.47	1.26	0.3	CHARMM36	POPC:SOL (1024:51200)	298.15	701
26	0.37	0.68	0.47	1.26	0.4	CHARMM36	POPC:SOL (200:8880)	300.00	164
27	0.34	0.66	0.45	1.26	0.3	CHARMM36	POPC:SOL (256:12800)	298.15	710
28	0.40	0.54	0.44	1.3	0.2	CHARMM36	SOL:POPE (5760:144)	310.00	430
29	0.34	0.65	0.44	1.46	0.2	CHARMM36	POPC:SOL (64:3200)	298.15	678
30	0.55	0.21	0.44	0.8	3.2	GROMOS-CKP	POPS:SOL:SOD (128:4480:128)	298.00	597
31	0.34	0.62	0.43	0.86	4.7	lipid17	POPC:SOL (128:5120)	298.15	30
32	0.34	0.58	0.42	1.56	0.7	CHARMM36	POPC:SOL (64:3200)	298.15	546
33	0.55	0.15	0.41	0.8	4.9	GROMOS-CKP	POPS:SOL:SOD (128:4480:128)	298.00	425
34	0.55	0.13	0.41	0.6	3.1	GROMOS-CKP	POPS:SOL:SOD (128:4480:128)	298.00	473
35	0.58	0.02	0.39	0.75	2.4	Berger/Höltje	POPC:CHOL:SOL (120:8:7290)	298.00	305
36	0.41	0.32	0.38	0.3	1.0	CHARMM36-UA	POPE:SOL (336:15254)	310.00	352
37	0.43	0.27	0.37	0.3	1.0	CHARMM36-UA	POPE:SOL (336:15254)	310.00	233
38	0.48	0.08	0.35	0.25	6.7	Orange	POPC:SOL (72:2880)	298.00	38
39	0.39	0.20	0.33	0.7	3.8	GROMOS-CKP	POPS:SOL:SOD (128:4480:128)	298.00	535
40	0.40	0.06	0.29	0.8	3.1	Charmm-Drude	POPE:SOL (144:5040)	310.00	731
41	0.33	0.17	0.28	0.76	5.8	Chiu Gromos	POPC:SOL (128:3552)	298.00	422
42	0.40	0.02	0.27	0.27	2.6	Berger/Höltje	POPC:CHOL:SOL (110:18:8481)	298.00	589
43	0.11	0.53	0.25	1.7	1.7	CHARMM36	SOL:POPE (25000:500)	310.00	67
44	0.29	0.12	0.23		5.6	Slipids	POPG:SOL:SOD (288:10664:288)	298.00	207
45	0.20	0.18	0.21	3.39	1.7	slipids	CHOL:POPC:SOL (256:256:20334)	298.00	82
46	0.25	0.07	0.19	1.36	1.0	GROMOS-CKP	POPC:SOL (500:25000)	298.00	202
47	0.24	0.09	0.19	1.6	1.3	Lipid17	POPE:SOL (500:25000)	310.00	195
48	0.21	0.11	0.18	0.3	2.5	Slipids	POPS:SOL:SOD (128:4480:128)	298.00	529
49	0.17	0.21	0.18	3.8	0.3	AMOEBA	POPE:SOL (72:2880)	310.00	730
50	0.12	0.26	0.17	0.8	3.0	CHARMM36-UA	POPS:SOL:SOD (128:4480:128)	298.00	263

Supplementary Figure 5. Top 50 simulations in the NMRlipids Databank ranked based on the C–H bond order parameter quality against experiments. The columns 2-4 show qualities for acyl chain order parameters (P^{tails}), headgroup order parameters (P^{hg}), all order parameters (P^{total}), and for X-ray scattering form factors (FF_q). Column 5 shows relative equilibration times for conformations (τ_{rel}). Note that the best possible order parameter quality is one, while the best possible form factor quality is zero. ID values in the last column can be used to identify each simulation in the databank.

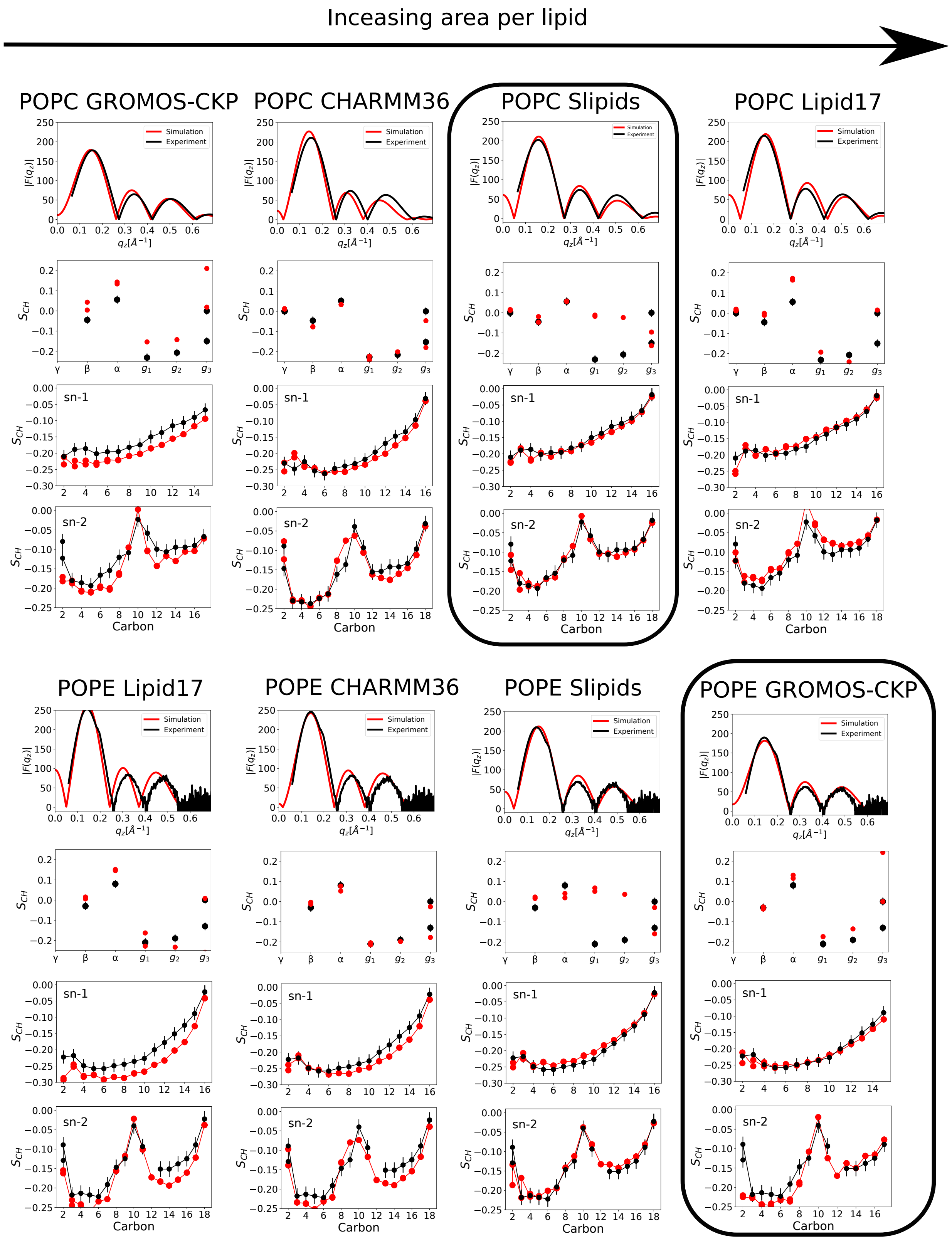

Supplementary Figure 6. Simulations with the data for both POPC (top) and POPE (bottom) directly compared with the experimental data. The area per lipid increases from left to right. Simulations with the best overall quality for POPC and POPE order parameters are highlighted with a solid border.

Testing machine learning models against literature data

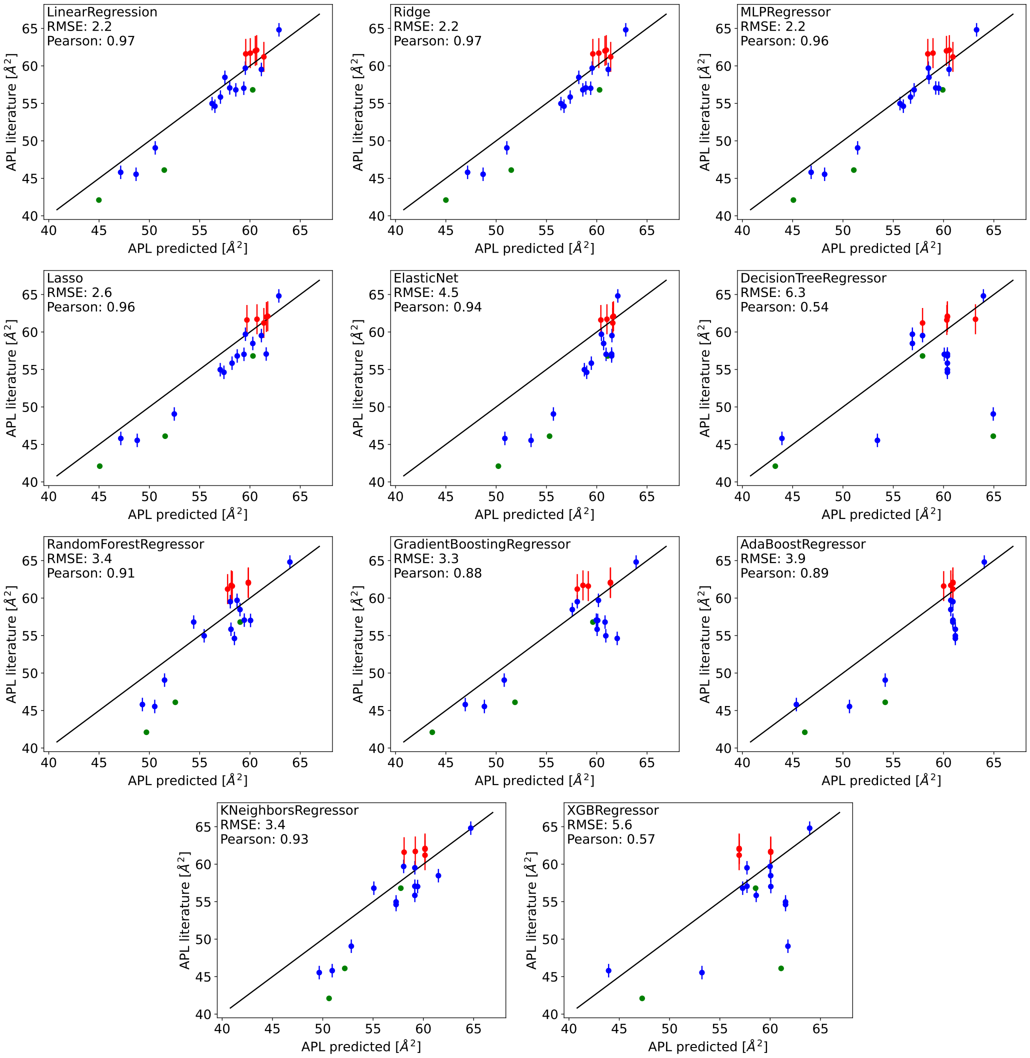

Supplementary Figure 7. Predictions of area per lipids of multi-component membranes composed of POPC, POPE, POPS, PI, sphingomyelin lipids and cholesterol from linear regression model against literature data from simulations using CHARMM36 parameters (green[31], blue[32], and red[33]). Mean squared error (RMSE) and Pearson correlation coefficient are shown for each model. The black line depicts x=y where the prediction equals the literature value. Our machine learning models that are trained using simulations with wide range of force field parameters have general tendency to predict larger area per lipid values than reported from CHARMM36 simulations in the literature. One explanation could be lower area per lipids predicted by the CHARMM36 parameters when compared with many other force fields in the training set[34], yet other explanations cannot be ruled out.

Water permeation through membranes

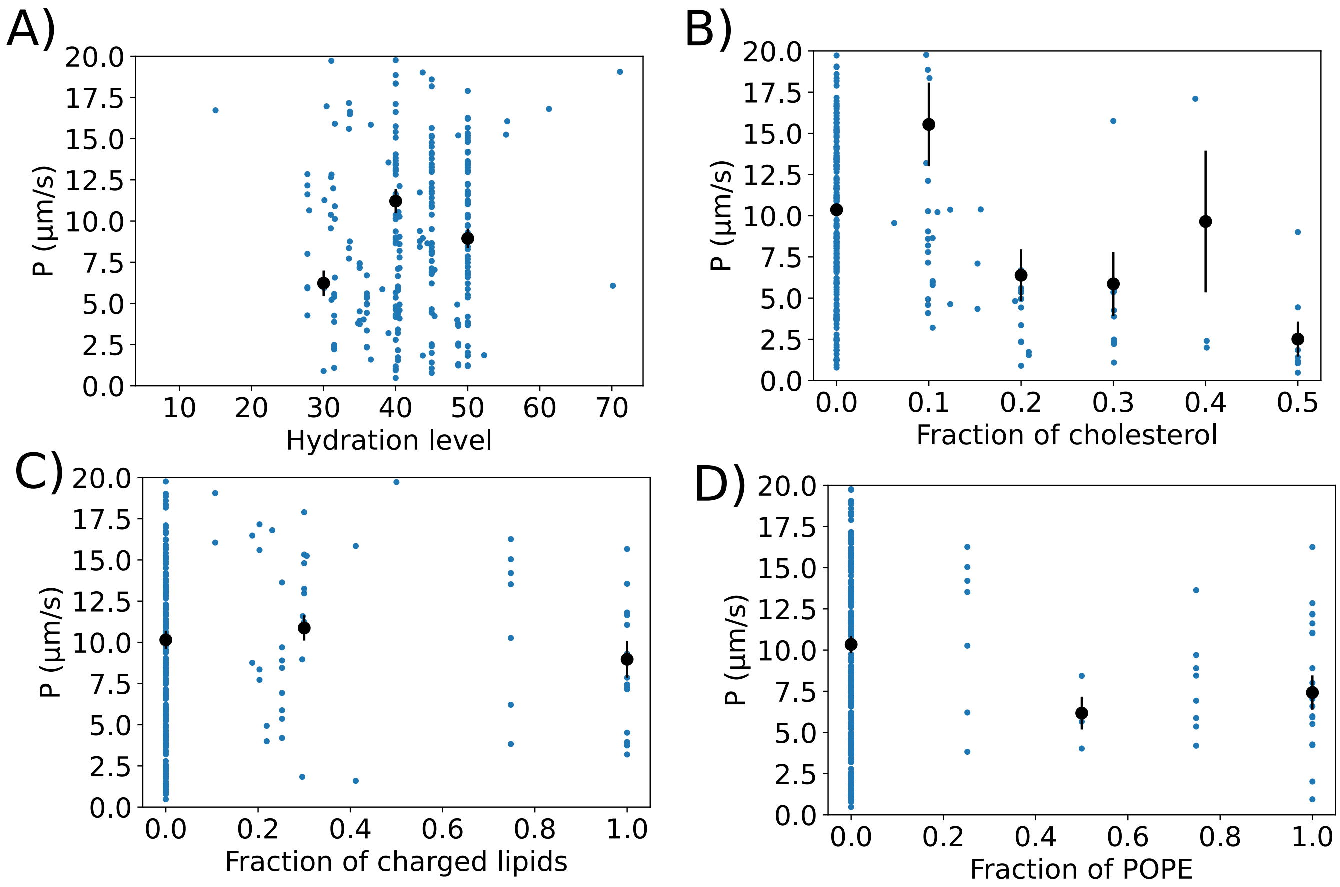

Supplementary Figure 8. Water permeation through membranes analyzed from the Databank as a function of (A) hydration level, (B) fraction of cholesterol, (C) fraction of charged lipids, and (D) fraction of POPE in membrane. Values from simulations with non-zero permeation values are shown with blue dots. Histogrammed values are shown with black dots For the mean value in each bin, average weighted with the simulation lengths was used, and error bars show the standard error of the mean. Only bins with more than one microsecond of data were used. Only simulations with the temperatures between 300-315 K were used.

Water diffusion along membranes

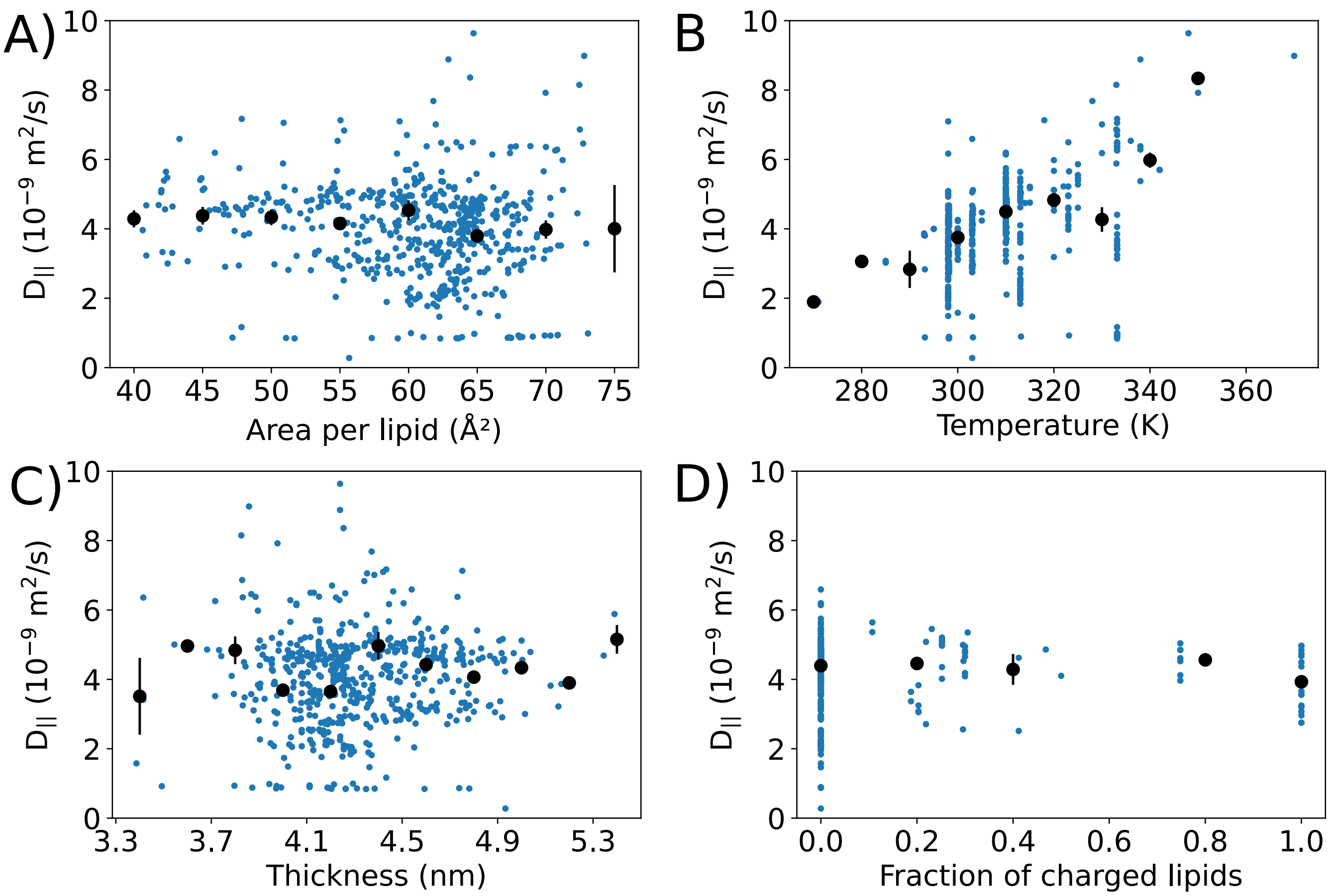

Supplementary Figure 9. Lateral diffusion of water as a function of (A) area per lipid, (B) temperature, (C) membrane thickness, and (D) fraction of charged lipids in a membrane. Non-zero permeation and diffusion values from simulations are shown with blue dots. Histogrammed values are shown with black dots. For the mean value in each bin, average weighted with the simulation lengths was used, and error bars show the standard error of the mean. Only bins with more than one microsecond of data in total were used for water permeation. Only simulations with the temperatures between 300-315 K were used in D.

NMR experiments

Acyl chain order parameters of POPE (Supplementary Figures 10 and 11) and POPG (Supplementary Figures 12 and 13) were analyzed from the same data that were previously recorded to determine headgroup order parameters[13]. The analysis of the crowded spectral region at 29–31 ppm was based on the previous assignment reported for POPC membranes[35]. To measure the order parameters for DOPC (Supplementary Figure 14), the sample was prepared and experiments performed similarly to previous studies[13].

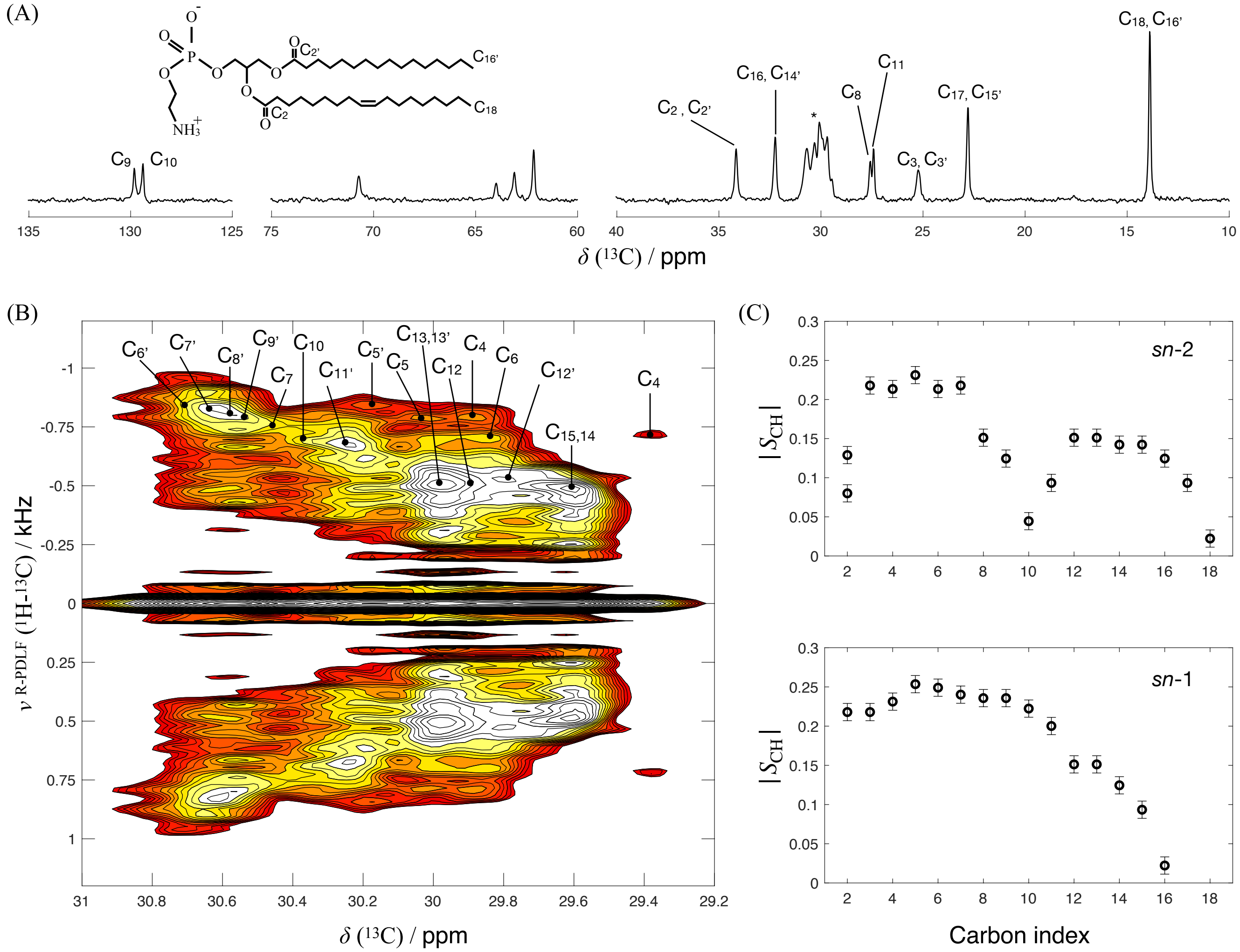

Supplementary Figure 10. Determination of the POPE acyl chain order parameters from a R-PDLF spectrum measured at a magic angle spinning frequency of 5.15 kHz. (A) ^{13}C rINEPT spectrum with peak assignment. The labels used are shown in the chemical structure of POPE. The chemical shift of the methyl groups was defined as 13.8 ppm. (B) Contour plot of the R-PDLF spectrum for the crowded spectral region. The assignment was based on a previous assignment reported for POPC membranes[35]. (C) C–H bond order parameter profile for the acyl chains of POPE. The splittings used for calculating the order parameters are shown in Supplementary Figure 11. The unassigned peaks belong to the headgroup and glycerol backbone carbons. A detailed assignment and order parameter analysis of these carbons was shown previously[13].

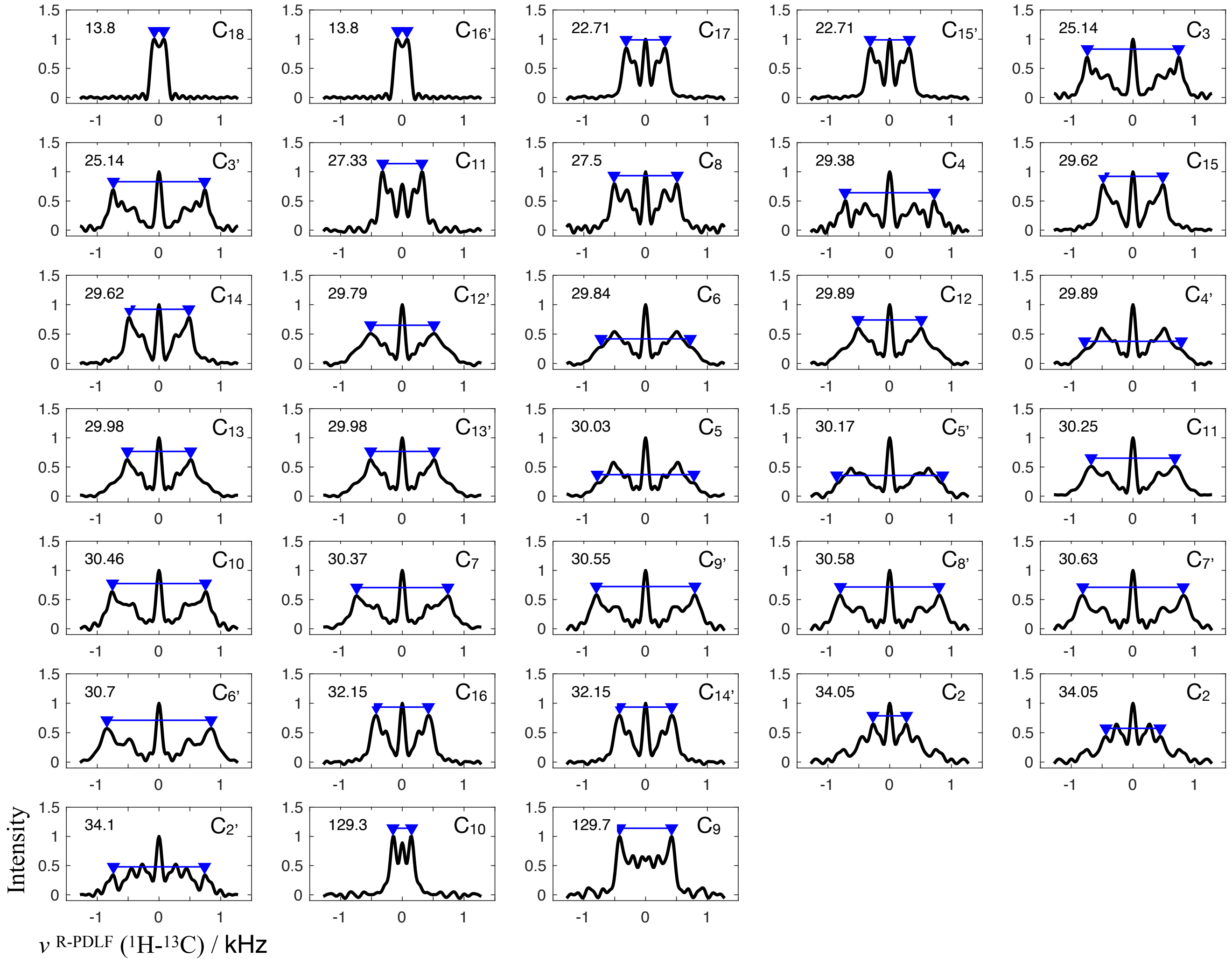

Supplementary Figure 11. Dipolar spectra obtained from the 2D R-PDLF spectrum from POPE in Supplementary Figure 10. The number at the top left corner of each panel denotes the corresponding chemical shift. The carbon label for each splitting is displayed on the top right corner. The labels are the same as in Supplementary Figure 10.

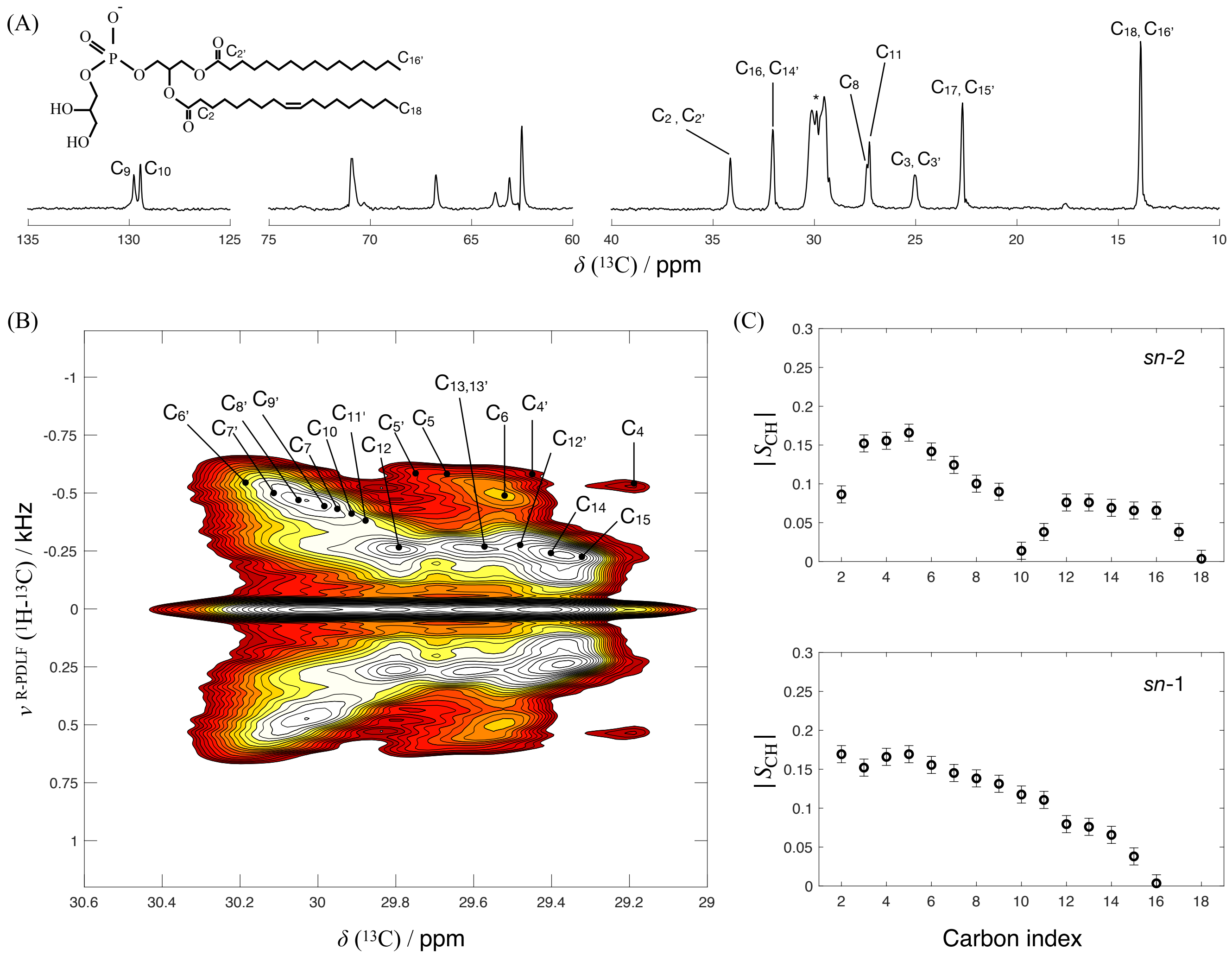

Supplementary Figure 12. Determination of the POPG acyl chain order parameters from a R-PDLF spectrum measured at a magic angle spinning frequency of 5.15 kHz. (A) ^{13}C rINEPT spectrum with peak assignment. The labels used are shown in the chemical structure of POPG. The chemical shift of the methyl groups was defined as 13.8 ppm. (B) Contour plot of the R-PDLF spectrum for the crowded spectral region. The assignment was based on a previous assignment reported for POPC membranes[35]. (C) C–H bond order parameter profile for the acyl chains of POPG. The splittings used for calculating the order parameters are shown in Supplementary Figure 13. The unassigned peaks belong to the headgroup and glycerol backbone carbons. A detailed assignment and order parameter analysis of these carbons was shown previously[13].

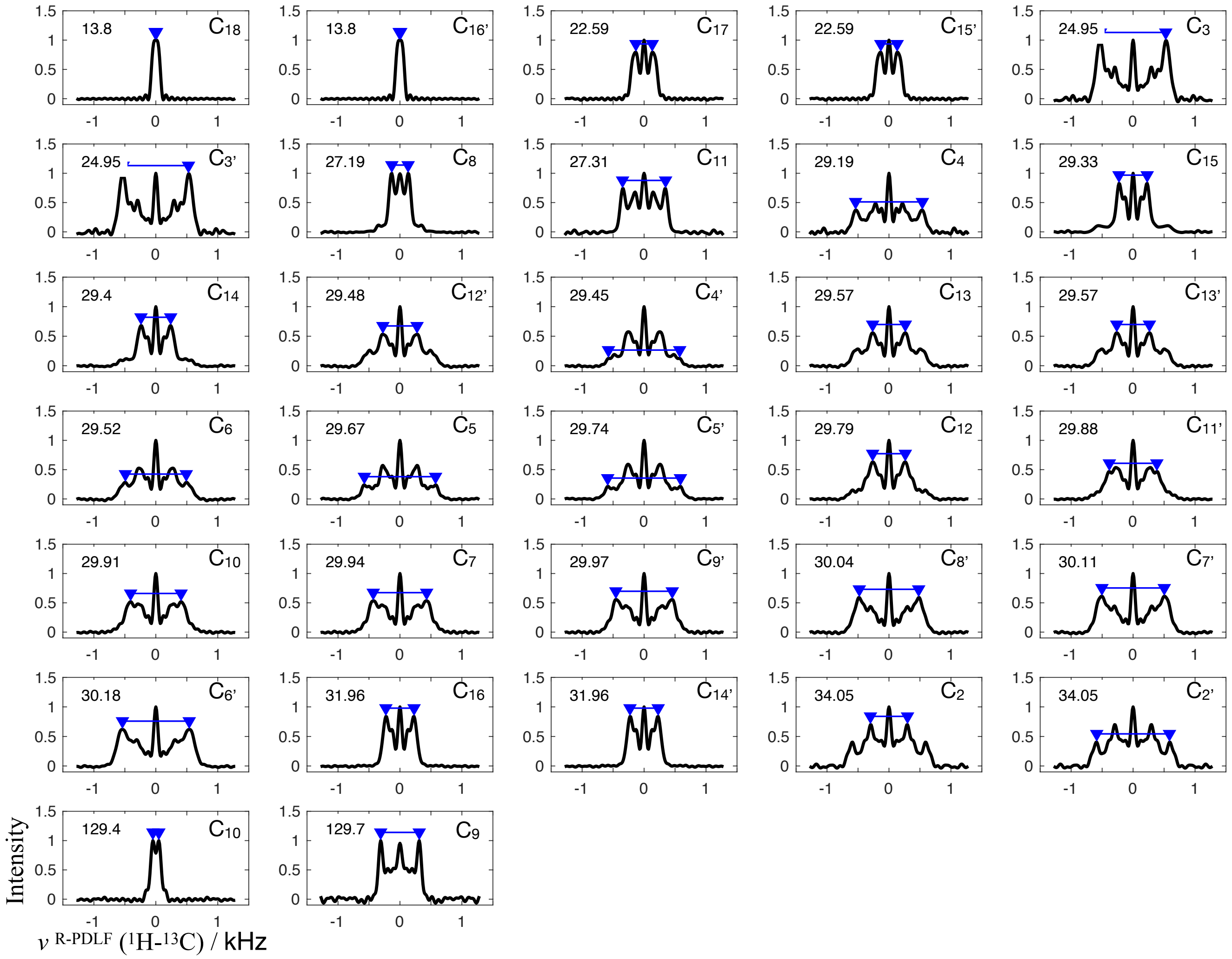

Supplementary Figure 13. Dipolar spectra obtained from the 2D R-PDLF spectrum described in Supplementary Figure 12. The number at the top left corner of each panel denotes the corresponding chemical shift. The carbon label for each splitting is displayed on the top right corner. The labels are the same as in Supplementary Figure 12.

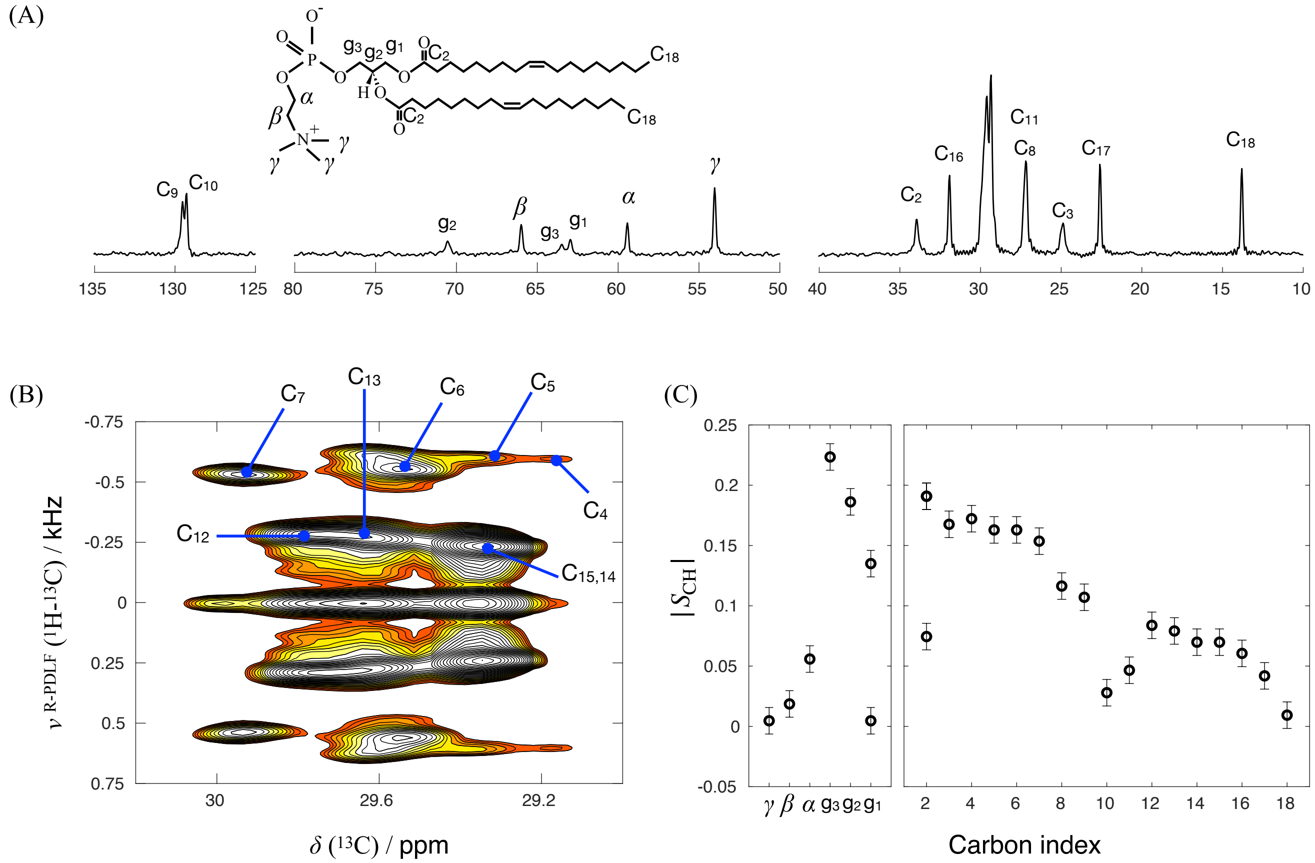

Supplementary Figure 14. Determination of DOPC order parameters from a R-PDLF spectrum measured at a magic angle spinning frequency of 5.15 kHz. (A) ^{13}C rINEPT spectrum with peak assignment. The labels used are shown in the chemical structure of DOPC. The chemical shift of the methyl groups was defined as 13.8 ppm. (B) Contour plot of the R-PDLF spectrum for the crowded spectral region. The assignment was based on a previous assignment reported for POPC membranes[35]. (C) C–H bond order parameters of DOPC.

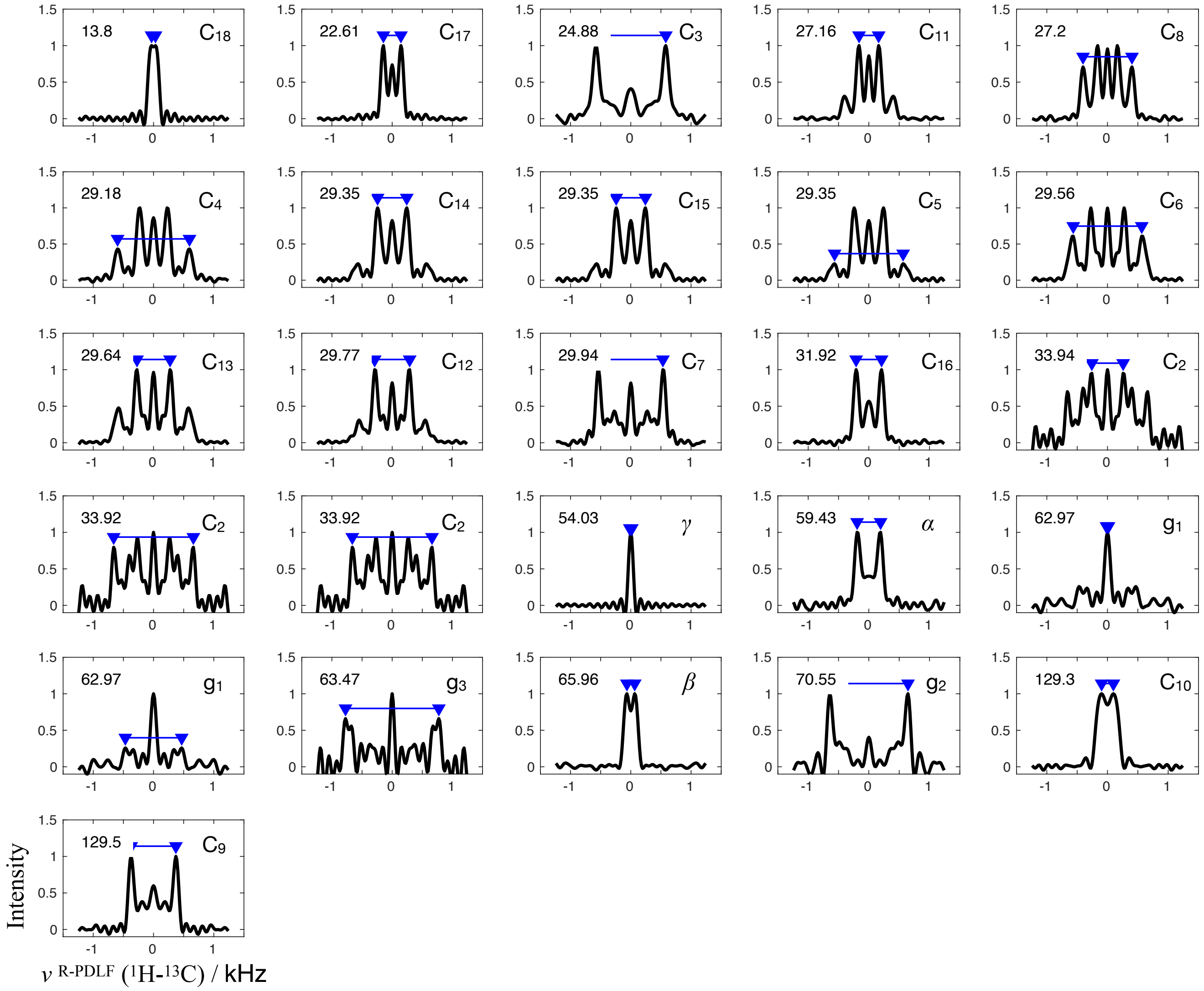

Supplementary Figure 15. Dipolar spectra obtained from the 2D R-PDLF spectrum described in Supplementary Figure 14. The number at the top left corner of each panel denotes the corresponding chemical shift. The carbon label for each splitting is displayed on the top right corner. The labels are the same as in Supplementary Figure 14.

Supplementary References

1. Klauda, J. B. *et al.* Update of the CHARMM all-atom additive force field for lipids: Validation on six lipid types. *J. Phys. Chem. B* **114**, 7830–7843 (2010).
2. Jämbeck, J. P. M. & Lyubartsev, A. P. Derivation and systematic validation of a refined all-atom force field for phosphatidylcholine lipids. *J. Phys. Chem. B* **116**, 3164–3179 (2012).
3. Jämbeck, J. P. M. & Lyubartsev, A. P. An extension and further validation of an all-atomistic force field for biological membranes. *J. Chem. Theory Comput.* **8**, 2938–2948 (2012).
4. Jämbeck, J. P. & Lyubartsev, A. P. Another piece of the membrane puzzle: extending slipids further. *J. Chem. Theo. Comput.* **9**, 774–784 (2012).
5. Ermilova, I. & Lyubartsev, A. P. Extension of the slipids force field to polyunsaturated lipids. *The J. Phys. Chem. B* **120**, 12826–12842 (2016).
6. Grote, F. & Lyubartsev, A. P. Optimization of slipids force field parameters describing headgroups of phospholipids. *J. Phys. Chem. B* **124**, 8784–8793 (2020).
7. Kulig, W., Pasenkiewicz-Gierula, M. & Róg, T. Cis and trans unsaturated phosphatidylcholine bilayers: A molecular dynamics simulation study. *Chem. Phys. Lipids* **195**, 12 – 20 (2016).
8. Dickson, C. J. *et al.* Lipid14: The amber lipid force field. *J. Chem. Theory Comput.* **10**, 865–879 (2014).
9. Dickson, C. J., Walker, R. C. & Gould, I. R. Lipid21: Complex lipid membrane simulations with amber. *J. Chem. Theo. Comput.* **18**, 1726–1736 (2022).
10. Li, H. *et al.* Drude polarizable force field for molecular dynamics simulations of saturated and unsaturated zwitterionic lipids. *J. chemical theory computation* **13**, 4535–4552 (2017).
11. Melcr, J. *et al.* Accurate binding of sodium and calcium to a popc bilayer by effective inclusion of electronic polarization. *J. Phys.Chem. B* **122**, 4546–4557 (2018).
12. Melcr, J., Ferreira, T. M., Jungwirth, P. & Ollila, O. H. S. Improved cation binding to lipid bilayers with negatively charged pops by effective inclusion of electronic polarization. *J. Chem. Theo. Comput.* **16**, 738–748 (2020).
13. Bacle, A. *et al.* Inverse conformational selection in lipid–protein binding. *J. Am. Chem. Soc.* **143**, 13701–13709 (2021).
14. Chandrasekhar, I. *et al.* A consistent potential energy parameter set for lipids: dipalmitoylphosphatidylcholine as a benchmark of the gromos96 45a3 force field. *Eur. Biophys. J.* **32**, 67–77 (2003).
15. Kukol, A. Lipid models for united-atom molecular dynamics simulations of proteins. *J. Chem. Theory Comput.* **5**, 615–626 (2009).
16. Piggot, T. J., Piñeiro, Á. & Khalid, S. Molecular dynamics simulations of phosphatidylcholine membranes: A comparative force field study. *J. Chem. Theory Comput.* **8**, 4593–4609 (2012).
17. Berger, O., Edholm, O. & Jähnig, F. Molecular dynamics simulations of a fluid bilayer of dipalmitoylphosphatidylcholine at full hydration, constant pressure, and constant temperature. *Biophys. J.* **72**, 2002 – 2013 (1997).
18. Nencini, R. *Development and testing of computer models of phospholipid membranes.* Master's thesis, Charles University of Prague, DOI: http://hdl.handle.net/20.500.11956/106127 (2019). Http://hdl.handle.net/20.500.11956/106127.
19. Catte, A. *et al.* Molecular electrometer and binding of cations to phospholipid bilayers. *Phys. Chem. Chem. Phys.* **18**, 32560–32569 (2016).
20. Poger, D., Van Gunsteren, W. F. & Mark, A. E. A new force field for simulating phosphatidylcholine bilayers. *J. Comput. Chem.* **31**, 1117–1125 (2010).
21. Chiu, S.-W., Pandit, S. A., Scott, H. L. & Jakobsson, E. An improved united atom force field for simulation of mixed lipid bilayers. *J. Phys. Chem. B* **113**, 2748–2763 (2009).
22. Dickson, C. J., Rosso, L., Betz, R. M., Walker, R. C. & Gould, I. R. GAFFlipid: a general amber force field for the accurate molecular dynamics simulation of phospholipid. *Soft Matter* **8**, 9617–9627 (2012).
23. Roos, K. *et al.* Opls3e: Extending force field coverage for drug-like small molecules. *J. Chem. Theory Comput.* **15**, 1863–1874 (2019).
24. Ulmschneider, J. P. & Ulmschneider, M. B. United atom lipid parameters for combination with the optimized potentials for liquid simulations all-atom force field. *J. Chem. Theory Comput.* **5**, 1803–1813 (2009).

25. Chu, H., Peng, X., Li, Y., Zhang, Y. & Li, G. A polarizable atomic multipole-based force field for molecular dynamics simulations of anionic lipids. *Molecules* **23**, 77 (2018).

26. Mobley, D. L. *et al.* Escaping atom types in force fields using direct chemical perception. *J. Chem. Theory Comput.* **14**, 6076–6092 (2018).

27. Boothroyd, S. *et al.* Development and benchmarking of open force field 2.0.0: The sage small molecule force field. *J. Chem. Theory Comput.* **19**, 3251–3275 (2023).

28. Openff-interchange. https://zenodo.org/doi/10.5281/zenodo.8147764. Accessed: 2013-10-21.

29. Santuz, H., Bacle, A., Poulain, P. & Fuchs, P. F. buildh: Build hydrogen atoms from united-atom molecular dynamics of lipids and calculate the order parameters. *J. Open Source Softw.* **6**, 3521 (2021).

30. Javanainen, M. Simulations of POPC/cholesterol mixtures at 298 K, three system sizes, CHARMM36, DOI: 10.5281/zenodo.7035350 (2021).

31. Shahane, G., Ding, W., Palaiokostas, M. & Orsi, M. Physical properties of model biological lipid bilayers: insights from all-atom molecular dynamics simulations. *J. Mol. Model.* **25**, 76 (2019).

32. Kumar, N. & Sastry, G. N. Study of lipid heterogeneity on bilayer membranes using molecular dynamics simulations. *J. Mol. Graph. Model.* **108**, 108000 (2021).

33. Oliveira, A. A. *et al.* Examining the effect of charged lipids on mitochondrial outer membrane dynamics using atomistic simulations. *Biomolecules* **12** (2022).

34. Javanainen, M. *et al.* Quantitative comparison against experiments reveals imperfections in force fields' descriptions of popc–cholesterol interactions. *J. Chem. Theory Comput.* **19**, 6342–6352 (2023).

35. Ferreira, T. M. *et al.* Cholesterol and POPC segmental order parameters in lipid membranes: solid state ^{1}H-^{13}C NMR and MD simulation studies. *Phys. Chem. Chem. Phys.* **15**, 1976–1989 (2013).
